# Measuring treatment attrition at various stages of engagement in Opioid Agonist Treatment in Ontario Canada using a cascade of care framework

**DOI:** 10.1186/s12913-022-07877-8

**Published:** 2022-04-12

**Authors:** Farah Tahsin, Kristen A. Morin, Frank Vojtesek, David C. Marsh

**Affiliations:** 1 University of Toronto, Toronto, Canada; 2 Northern Ontario School of Medicine, Sudbury, ON P3E 2C6 Canada; 3 ICES North, Sudbury, Canada; 4 Health Sciences North Research Institute, Sudbury, Canada; 5 Canadian Addiction Treatment Centres, Markham, Canada

**Keywords:** Opioid use disorder, Opioid agonist treatment, Cascade of care, Treatment attrition

## Abstract

**Background:**

The cascade of care framework is an effective way to measure attrition at various stages of engagement in Opioid Agonist Treatment (OAT). The primary objective of the study was to describe the cascade of care for patients who have accessed OAT from a network of specialized addiction clinics in Ontario, Canada. The secondary objectives were to evaluate correlates associated with retention in OAT at various stages and the impact of patients’ location of the residence on retention in OAT.

**Design:**

A multi-clinic retrospective cohort study was conducted using electronic medical record (EMR) data from the largest network of OAT clinics in Canada (70 clinics) from 2014 to 2020. Study participants included all patients who received OAT from the network of clinics during the study period.

**Measurements:**

In this study, four stages of the cascade of care framework were operationalized to identify treatment engagement patterns, including patients retained within 90 days, 90 to 365 days, one to 2 years, and more than 2 years. Correlates associated with OAT retention for 90 days, 90 to 365 days, 1 to 2 years, and more than 2 years were also evaluated and compared across rural and urban areas in northern and southern Ontario.

**Results:**

A total of 32,487 patients were included in the study. Compared to patients who were retained in OAT for 90 days, patients who were retained for 90 to 365 days, 1 to 2 years, or more than 2 years were more likely to have a higher number of treatment attempts, a higher number of average monthly urine drug screening and a lower proportion of positive urine drug screening results for other drug use.

**Conclusion:**

Distinct sociodemographic and clinical factors are likely to influence treatment retention at various stages of engagement along the OAT continuum. Research is required to determine if tailored strategies specific to people at different stages of retention have the potential to improve outcomes of OAT.

## Introduction

There is a growing burden of disease due to opioid-related morbidity and mortality in North America [1]. According to the Public Health Agency of Canada [2], from January 2016 to March 2021, 22,828 individuals died from apparent opioid toxicity. Since 2016, the province of Ontario has experienced an upward trend in opioid-related deaths. For example, in 2020, there were 2430 opioid-related deaths (16.5 per 100,000 population per year), an increase from 2016 when there were 867 (6.2 per 100,000 population) deaths for the entire year. Of note, over the course of the Covid-19 pandemic, the opioid-related health crisis worsened in Ontario due to an increased sense of isolation, stress, and anxiety and limited availability of support services for patients who use opioids [2]. All these numbers combined demonstrate a growing concern for Opioid Use Disorder (OUD) in Ontario.

OUD is a chronic condition that requires ongoing engagement with treatment [3]. However, due to the wide array of clinical, health system, and socio-demographic factors, poor retention and dropout are common among patients with OUD [4–6]. For example, lack of patient education related to treatment options, receiving care from outpatient clinics, a mismatch between patient-provider treatment goals, and perceived difficulty of withdrawal may cause patients to withdraw and/or stop OUD treatment [4]. Additionally, socio-economic factors such as low family income, living in deprived neighborhoods such as neighborhoods with high crime rates and residents with lower socio-economic status, and history of homelessness can also cause poor retention [5]. This poor retention and frequent stopping can often be fatal [7]. Opioid Agonist Treatment (OAT) and subsequent retention in OAT have been proven to be the most effective intervention to manage OUD [3, 8, 9]. In Ontario, most patients with OUD receive care in specialized OAT clinics [10]. Specialized OAT clinics in Ontario are privately run clinics operating under a fee-for-service model of care. For Ontario residents, physician services are paid for publicly by the Ontario Health Insurance Plan (OHIP), and medications are paid for out of pocket or by private insurance from the patient. The majority of Ontario residents are eligible for public drug coverage if they are aged 65 years or older, reside in a long-term care facility, are disabled, are receiving social benefits for income support, or have high prescription drug costs relative to their net household income. The Canadian Addiction Treatment Centers (CATC) is the largest network of addiction medicine clinics in Canada (approximately 70 clinics across Ontario). CATC provides comprehensive care for patients with OUD, which includes Methadone and Buprenorphine/Naloxone assisted therapy, primary care, harm reduction, and counseling [11]. Standardized practices, policies, and operating procedures within the clinic network, limit the likelihood of treatment variability between sites.

As of 2021, only physicians and nurse practitioners can prescribe Methadone. However, a nurse or pharmacist could supervise observed daily dosing during treatment stabilization [12]. In Ontario, Prescribing Buprenorphine/Naloxone and Methadone requires a written or faxed prescription from a prescriber who is expected to have undergone appropriate training on treatment and addiction medicine. There are important differences between Methadone and Buprenorphine/naloxone. At the time of this study, the Canadian Research Initiative in Substance Misuse developed *National Guidelines for the Clinical Management of Opioid Use Disorder* recommended using buprenorphine/naloxone as the first-line therapy [13]. Buprenorphine/naloxone is recognized as an ideal first-line agent relative to methadone because it is associated with the following benefits: fewer regulatory prescription barriers, fewer drug-to-drug interactions and, less adverse effects such as respiratory depression and QT prolongation [13].

There is regional variation in the availability of OAT prescribers in Ontario. For example, individuals living in rural and northern areas in Ontario have less access to specialized services and must travel further to access OAT [14]. One reason behind this regional difference is that the population distribution in Northern and Southern Ontario is vastly different. According to the 2006 census, only 6% of the Ontario population lives in Northern Ontario, whereas 94% lives in Southern Ontario [12]. Additionally, it is well established in the literature that there are notable differences in characteristics between patients living in Northern and Southern Ontario including rurality, higher chronic diseases and smoking rates, older age, lower socio-economic status, and less access to health services [15–17].

The literature has shown that one-year retention in OAT is associated with positive outcomes, including reduced mortality rates, reduced drug use, reduced infections and high-risk drug use behaviors causing overdoses, reduced crime, improved psychosocial relationships, and increased employment rates [18, 19]. However, previous studies have shown that patients with OUD often only stay in treatment for 30–60 days [20, 21] which may be reducing the effectiveness of OAT. Moreover, reports have shown low engagement for people with OUD in the current treatment system in Ontario [22]. For example, Ontario Drug Policy Research Network (ODPRN) is an established research network that tracks trends and patterns of drug utilization, safety, and utilization in Ontario [22]. The ODPRN reported that, in 2015, there were a total of 259,674 people who were prescribed opioids in Ontario (7133/10,000 Ontario Drug Benefit (ODB) Program Eligible), while only 33,693 patients were currently in OAT (927/per 10,000 ODB eligible) [22]. This shows that a vast majority of patients were not engaging with the treatment system [22].

The cascade of care framework is an effective way to report patients’ engagement and attrition from a treatment system [11]. The key objective of a cascade of care framework is to measure patients’ engagement at the critical stages of treatment, such as engagement in care, initiation of medication, and subsequent retention [23]. By tracking these key stages of the treatment system, we can better understand care fragmentation and subsequently target policy and clinical intervention to bridge the care gaps. The cascade of care has been used to measure engagement patterns for other chronic conditions that require ongoing support and management, such as diabetes, HIV infection, chronic Hepatitis C infection, and OUD [3, 24–27].

The cascade of care framework has been proposed to guide the public health responses towards the opioid crisis, given patients with OUD receive more significant benefits from prolonged engagement with OAT [24, 28]. Several cascades of care studies for OUD have been published across North America [24, 29, 30]. However, no such data has been published for Ontario, Canada. Additionally, the literature relating to geographical variation of OAT engagement and retention is limited [31, 32]. Therefore, the objectives for the current study is to 1) describe the cascade of care for patients who have accessed OAT from a network of specialized addiction clinics in Ontario, Canada; 2) evaluate correlates associated with retention in OAT at various stages along the continuum of engagement; 3) assess the impact of patients’ location of the residence on retention in OAT.

## Methods

### Study design

We conducted a retrospective study utilizing electronic medical records (EMR) from January 2014 to 2020. A total of 32,487 adults from the CATC in Ontario, Canada, were included in the study. We followed patients from the first record of OAT prescription (including methadone or buprenorphine/naloxone) to administrative loss to follow up. Administrative loss to follow-up was defined when a treatment window ended, and no other treatment window was started by the end of the study window.

The study data was accessed remotely using a secure server. Patient identification was anonymized by removing names and health card numbers. The Laurentian University Research Ethics Board provided ethical approval for this study. The Strengthening the Reporting of Observational Studies in Epidemiology (STROBE) guidelines were used to write this manuscript [33].

### Study setting

All OAT prescriptions for patients in this study were captured for analysis regardless of eligibility for publicly funded drug coverage. We have defined OAT as “the treatment that involves forms of methadone and buprenorphine/naloxone as a treatment method”. Since slow-release oral morphine and injectable OAT are less frequently used in Ontario, those were not included in this study.

### Key measures

#### The OUD cascade of care

We defined four stages of OUD cascade care, focused on treatment initiation to long-term retention in OAT. The stages of the cascade of care included retention in OAT for 90 days; more than 90 days but less than a year; 1 to 2 years and, more than 2 years. The end of an episode was defined when patients had 5 days without a methadone dose and 6 days without a buprenorphine dose in accordance with clinical guidelines indicating the need to re-initiate clients on starting doses following absences of these durations [34].

#### Demographics and clinical history

We described patients who accessed OAT in the clinic network according to covariates known to influence engagement in OAT [35–37]. Covariates encompassed demographic and clinical factors. Demographic information included: age, region of health service delivery sex (male or female). The Ontario Medical Association (OMA) online Rurality Index of Ontario (RIO) score matching application program interface (API) was used to check RIO scores to postal codes. The health care at home API was used to corroborate Local Health Integration Network (LHIN) scores to postal codes [38]. The LHIN were the regional health authorities responsible for the regional administration of public healthcare services in Ontario [39]. LHIN was created to enable regional administration of healthcare services. Ontario had 14 LHINs that provided hospital and community-based care to all residents within their geographical boundaries [39]. Patients with missing postal codes (*n* = 4735) could not be included in the geographical analysis. Therefore, a subgroup analysis was conducted on a subset of the cohort (*n* = 27,939 patients). Patients were divided into four geographical regions for the subgroup analysis: Southern urban, Southern rural, Northern urban, and Northern rural. Northern regions were defined by LHIN 13 and 14. The North/South divide has been used in several peer review studies and reports [32, 40, 41]. Rural regions were defined as any region with an RIO score of 40 or higher [42].

Clinical factors included: initial OAT medication (Methadone or Buprenorphine/Naloxone), the longest number of days retained in OAT, starting and peak methadone dose, starting and peak buprenorphine/naloxone dose, and urine drug screening (UDS) results for cocaine, fentanyl, cannabis, and all opioids other than fentanyl and the patient’s OAT medication. UDS groups were created based on the proportion of positive UDS for each drug and divided into quadrants 0–25%, 26–50%, 51–75%, and 76–100%. The frequency of UDS is very consistent between physicians but there is an allowance for patient-specific variation based on clinical judgment. Take-home doses are linked to drug-free urines in an explicit contingency management schedule which physicians and patients review frequently together. UDS results were obtained using The FaStep Assay (Trimedic Supply Network Ltd., Concord, Ontario, Canada) with results for assays detecting amphetamine or methamphetamine combined for amphetamine-type stimulant results and assays detecting morphine or oxycodone combined for other opioid results. Results for fentanyl, cannabis, and cocaine are based on specific assays detecting fentanyl, THC, and cocaine metabolites.

### Statistical analysis

We first plotted the OAT cascade of care from 2014 to 2020 and provided population characteristics. We then conducted a multinomial regression model from 2014 to 2020. Our response variable was four stages of a cascade of care (< 90 days, 90–365 days, 1 to 2 years, and more than 2 years). We identified 32,663 patients who received OAT in CATC clinics across Ontario from 2014 to 2020. However, we analyzed data for 32,487 because *n* = 176 patients had key missing variables, so they were excluded from the analysis. Analysis was conducted using R version 4.0.1. An alpha level of 0.05 was used for all statistical tests.

## Results

### The OUD cascade of care

**Fig. 1:**
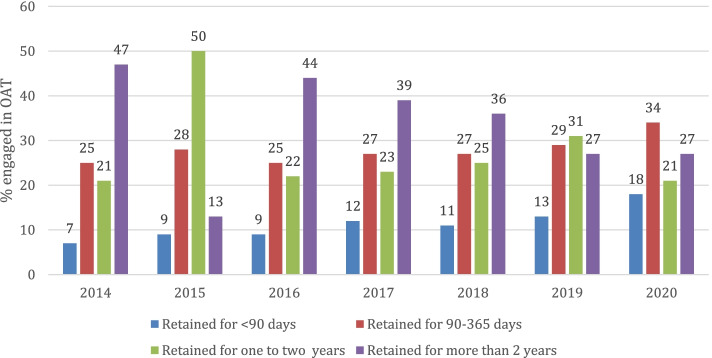
The Opioid Use Disorder Cascade of Care in Ontario, Canada, 2014–2020

A total of 32,487 patients were included in the analysis. The OUD Cascade of care is plotted in Fig. 1. The total number of patients who restarted in OAT increased substantially from 1867 in 2014 to 4575 in 2020. In 2014, 47% (873/1867) patients were retained in OAT for more than 2 years, whereas in 2020, 27% (1258/4575) patients were retained for more than 2 years.

### Characteristics of patients with opioid use disorder in Ontario, Canada, 2014–2020

Cohort characteristics are described in Table 1 and compared across treatment cascades. A total of 6087 (18.73%) patients were retained for less than 90 days of OAT, 7247 (22.31%) were retained for 90–365 days, 5, 413 (40.13%) patients were retained for 1 to 2 years, and 13, 740 or 42.49% were retained by more than 2 years. The overall mean age of the patients was 35.60 (SD = 10.70), and 61.4% of patients were male. We observed that 25,218 (77.60%) patients received methadone as a starting medication, and 7269 (22.40%) patients received buprenorphine/naloxone. The overall average treatment attempts were 2.17 (SD = 2.40).

**Table 1:** Characteristics of patients who received opioid agonist treatment from the Canadian Addiction Treatment Center in Ontario, Canada, 2014–2020

	Less than 90 days	90 to 365 days	One to two years	Over two years	Overall	***p***-value
	*n* = 6087 (18.73%)	*n* = 7247 (22.31%)	*n* = 5413 (40.13%)	*n* = 13,740 (42.49%)	*N* = 32,487	
**Gender**						
F n (%)	2227 (36.60)	27,07 (37.40)	2080 (38.40)	5485 (39.90)	12,499 (38.50)	<.001
M	3833 (63.00)	4535 (62.50)	3331 (61.50)	8255 (60.10)	19,949 (61.40)	
Missing	27 (0.40)	10 (0.10)	2 (0%)	0	41 (0.10)	
**Age**						
Mean (SD)	34.40 (10.50)	35.00 (10.51)	35.50 (10.80)	36.50 (10.72)	35.60 (10.70)	
**Age Groups**						<.001
< 25	1247 (20.50)	1318 (18.20)	1022 (18.90)	2116 (15.40)	5703 (17.60)	
25–45	3855 (63.30)	4725 (65.20)	3384 (62.50)	8591 (62.50)	20,653 (63.20)	
46–65	949 (15.60)	1147 (15.80)	970 (17.90)	2980 (21.70)	6046 (18.70)	
65+	36 (0.60)	57 (0.80)	37 (0.70)	53 (0.40)	183 (0.60)	
**Starting medication (mg)**						<.001
Methadone	4101 (67.40)	5171 (71.40)	4222 (78.00)	11,724 (85.30)	25,218 (77.60)	
Buprenorphine/naloxone	1986 (32.60)	2077 (28.60)	1191 (22.00)	2016 (14.70)	7269 (22.40)	
**Location of residence**						<.001
Northern/rural	183 (3.00)	310 (4.30)	235 (4.30)	675 (4.90)	1403 (4.30)	
Northern/urban	9,46 (15.50)	1275 (17.60)	915 (16.90)	2685 (19.50)	5821 (17.90)	
Sothern/rural	171 (2.80)	213 (2.90)	196 (3.60)	525 (3.80)	1101 (3.40)	
Southern/urban	4787 (78.760)	5449 (75.20)	4067 (75.10)	9855 (71.70)	24,158 (74.40)	
**No. of Treatment Attempts**						
Mean (SD)	1.87 (2.03)	2.67 (3.08)	2.67 (2.92)	1.86 (1.77)	2.17 (2.40)	<.001
**Average Monthly Urine Drugs Screening (UDS)**						
Mean (SD)	5.24 (2.59)	6.70 (1.93)	6.42 (1.71)	5.91 (1.54)	6.05 (1.96)	<.001
**Stimulant UDS positive groups (n%)**						<.001
**0–25%**	4373 (71.80)	5438 (75.00)	4323 (79.90)	11,749 (85.50)	25,883 (79.70)	
**26–50%**	286 (4.70)	582 (8.00)	397 (7.30)	835 (6.10)	2100 (6.50)	
**51–75%**	432 (7.10)	482 (6.70)	307 (5.70)	538 (3.90)	1759 (5.40)	
**76–100%**	996 (16.40)	745 (10.30)	386 (7.10)	618 (4.50)	2745 (8.40)	
**Fentanyl UDS positive groups (n%)**						<.001
**0–25%**	4368 (71.80)	5366 (74.10)	4293 (79.30)	12,101 (88.10)	26,128 (80.40)	
**26–50%**	218 (3.60)	578 (8.00)	481 (8.90)	890 (6.50)	2167 (6.70)	
**51–75%**	440 (7.20)	572 (7.90)	323 (6.00)	476 (3.50)	1811 (5.60)	
**76–100%**	1061 (17.40)	731 (10.10)	316 (5.80)	273 (2.00)	2381 (7.30)	
**Cocaine UDS positive groups (n%)**						<.001
**0–25%**	3246 (53.30)	4179 (57.70)	3562 (65.80)	10,163 (74.00)	21,149 (65.10)	
**26–50%**	684 (11.20)	794 (14.70)	794 (14.70)	1765 (12.80)	4358 (13.30)	
**51–75%**	712 (11.70)	849 (11.70)	525 (9.70)	943 (6.90)	3029 (9.30)	
**76–100%**	1445 (23.70)	1106 (15.03)	533 (9.80)	869 (6.30)	3953 (12.20)	
**Cannabis UDS positive groups (n%)**						<.001
**0–25%**	4709 (77.40)	3983 (55.00)	2455 (45.40)	5690 (4.40)	25,883 (79.70)	
**26–50%**	78 (1.30)	377 (5.20)	413 (7.60)	1275 (9.30)	2100 (6.50)	
**51–75%**	202 (3.30)	496 (6.80)	417 (5.70)	1114 (8.10)	1759 (5.40)	
**76–100%**	1098 (18.00)	2391 (33.00)	2128 (39.30)	5665 (41.20)	2745 (8.40)	
**Other Opioids UDS positive groups (n%)**						<.001
**0–25%**	3261 (53.60)	5042 (69.60)	4287 (79.20)	11,889 (86.50)	24,479 (75.40)	
**26–50%**	1136 (18.70)	1330 (18.30)	787 (14.50)	1373 (10.00)	4626 (14.20)	
**51–75%**	894 (14.70)	643 (8.00)	262 (4.80)	396 (2.90)	2206 (6.80)	
**76–100%**	796 (13.10)	232 (3.20)	77 (1.40)	82 (0.60)	1187 (3.70)	
**Starting BUP Dose (mg)**						
Mean (SD)	7.71 (7.91)	9.93 (13.50)	12.4 (18.30)	15.4 (21.48)	11.8 (16.88)	<.001
**Peak BUP dose (mg)**						
Mean (SD)	12.6 (10.10)	19.4 (32.55)	22.5 (21.41)	26.1 (23.90)	20.7 (24.50)	<.001
**Starting Methadone dose (mg)**						
Mean (SD)	33.3 (26.50)	39.9 (123)	43 (44.40)	57.7 (41.90)	47.4 (91.7)	<.001
**Peak Methadone Dose (mg)**						
Mean (SD)	51.6 (55.2)	86.5 (285)	99 (304)	119 (369)	97.5 (309)	<.001
**Start dose above-median peak dose (mg)**						
Mean (SD)	0.22 (0.41)	0.33 (0.47)	0.41 (0.49)	0.56 (0.49)	0.42 (0.49)	<.001
**Peak dose above-median peak dose (mg)**						
Mean (SD)	0.08 (0.27)	0.13 (0.33)	0.17 (0.38)	0.29 (0.45)	0.20 (0.40)	<.001

Abbreviations in the table: ^a^*SD* Standard deviation, *UDS* Urine Drug Screening, *BUP* Buprenorphine

### The cascade of care by geographical location

**Fig. 2:**
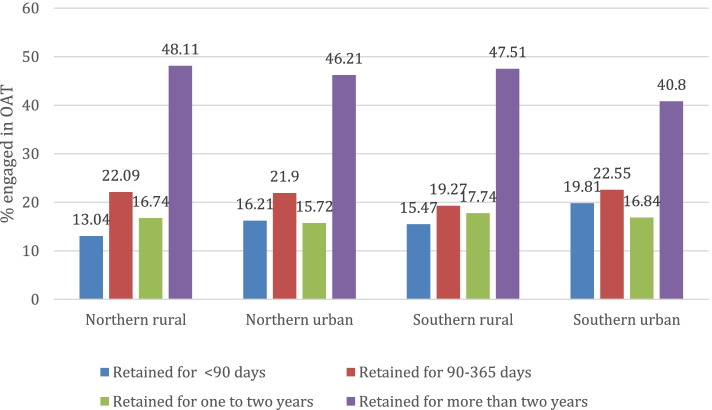
Regional variation in the treatment retention cascade of care for patients who received opioid agonist treatment (OAT) from the Canadian Addiction Treatment Center in Ontario, Canada, 2014–2020

The population distribution in Northern and Southern Ontario is vastly different. According to the 2006 census, only 6% of the Ontario population lives in Northern Ontario, whereas 94% lives in Southern Ontario [16]. A total of 1403 (4.3%) patients in the study cohort lived in a Northern rural area, whereas 5821(17.9%) lived in a Northern urban area, 1105 (3.4%) lived in Southern rural, and 14,158 (74.4%) lived in Southern urban area. Figure 2 shows the regional variation in the treatment retention cascade of care. Overall, a similar percentage of patients with OUD from four geographical regions were retained in each cascade of care. However, across all four regions, greater retention was observed in the cascade of more than 2 years.

### Cascade attrition after diagnosis and treatment engagement

Compared to patients who were retained for less than 90 days, patients who were retained in the OAT within 90 to 365 days were less likely to have methadone as starting medication [aOR = 0.80, 95% CI: 0.64–1.00], had a higher number of treatment attempts [aOR = 1.15, 95% CI: 1.04–1.18], had a higher frequency of UDS tests [aOR = 1.52, 95% CI: 1.42–1.61]. Additionally, compared to patients who were retained for less than 90 days, those retained in the OAT for 90 to 365 days were less likely to have frequent drug use, including amphetamine-type stimulants, fentanyl, cocaine, cannabis, and other opioids as measured by percent-positive UDS results.

Compared to patients who were retained in the OAT for less than 90 days, patients who were retained for 1 to 2 years were older, had a higher number of treatment attempts [aOR = 1.17, 95% CI: 1.15–1.19], had a higher frequency of monthly UDS tests [aOR = 1.56, 95% CI: 1.46–1.67], but less likely to have methadone as starting medication [aOR = 0.61, 95% CI: .48–.78]. Patients retained for 1 to 2 years were also less likely to have frequent drug use, including amphetamine-type stimulants, fentanyl, cocaine, cannabis, and other opioids as measured by percent-positive UDS results, compared to those retained in the OAT for less than 90 days.

Compared to patients who were retained in OAT for less than 90 days, patients who were retained for more than 2 years were more often under the age group of 65, lived in Southern rural and Northern urban region, had a higher frequency of monthly UDS [aOR = 1.33, 95% CI: 1.25–1.40], and had starting dose above-median [aOR = 1.40, 95% CI: 1.06–1.87]. Patients who were retained for more than 2 years compared to a shorter cascade of fewer than 90 days were less likely to have methadone as starting medication [aOR = 0.50, 95% CI: .40–.63]. Patients who were retained for over 2 years were also less likely to have frequent drug use, including amphetamine-type stimulants, fentanyl, cocaine, and other opioids as measured by percent-positive UDS results, compared to patients who were retained in the OAT for 90 days. However, they were more likely to use cannabis frequently. Detailed results are presented in Table 2.

**Table 2:** Crude and adjusted multinomial regression analysis of demographic factors associated with OAT engagement in Ontario Canada, 2014–2020

Variables (Ref = Less than 90 days^a^)	90 to 365 days		One to two years		Over two years	
	OR [95% CI]	aOR [95% CI]	OR [95% CI]	aOR [95% CI]	OR [95% CI]	aOR [95% CI]
**Female (ref: male)**						
	1.02 [0.95–1.10]	0.84 [0.68–1.03]	1.07 [.99–1.15]^a^	0.94 [0.75–1.19]	1.14 [1.07–1.21]	1.13 [.91–1.39]
**Age Groups (ref:** 65**+)**						
< 25	0.68 [0.45–1.00]	1.6 [.64–4.19]	0.82 [.52–1.30]	4.60 [1.78–11.88]^a^	1.22 [.80–1.9]	5.37 [2.20–13.13]^a^
25–45	0.79 [0.53–1.21]	1.62 [0.67–3.90]	0.88 [0.56–1.40]	4.30 [1.71–10.77	1.60 [1.06–2.40]	5.96 [2.54–13.9]^b^
46–65	0.77 [0.51–1.21]	1.30 [0.51–3.31]	1.00 [0.64–1.60]	3.24 [1.16–9.05]^a^	2.21 [1.46–3.40]^b^	4.67 [1.82–11.95]^a^
**The geographical location (ref: Southern urban)**						
Southern rural	1.12 [0.92–1.3]	1.07 [0.61–1.86]	0.67 [0.20–2.35]	1.20 [0.71–2.35]	1.50 [1.28–1.82]	1.97 [1.15–3.97]^a^
Northern urban	1.22 [1.08–1.3]	0.99 [0.75–1.29]	1.10 [1.03–1.26]	0.88 [0.66–1.76]	1.40 [1.27–1.50]	1.09 [1.15–3.39]^a^
Northern rural	1.50 [1.24–1.79]^a^	0.92 [0.56–1.50]	1.50 [1.24–1.84]^a^	0.94 [0.56–1.57]	1.80 [1.52–2.12]	1.29 [0.79–2.09]
**Methadone Starting medication (ref: Buprenorphine/naloxone)**						
	0.81 [0.75–.87]^a^	0.80 [0.64–1.01]^a^	0.57 [0.52–.61]^a^	0.61 [0.48–0.78]^a^	0.35 [0.32–0.37]^a^	0.50 [0.40–0.63]^a^
**Number of treatment attempts**						
	1.09 [1.15–1.19]^a^	1.15 [1.04–1.18]^a^	1.20 [1.15–1.19]^a^	1.17 [1.13–1.21]^a^	1.00 [0.99–1.02]	1.01 [0.96–1.04]
**Average monthly UDS**						
	1.52 [1.48–1.54]^a^	1.52 [1.42–1.61]^a^	1.40 [1.37–1.43]^a^	1.56 [1.46–1.67]^a^	1.21 [1.19–1.23]	1.33 [1.25–1.40]^a^
**Amphetamine-type stimulant UDS Groups (ref: 0–25%)**						
25–50%	1.70 [1.15–1.25]	0.83 [0.59–1.17]	1.46 [1.24–1.71]^a^	0.65 [0.46–0.96]^a^	1.13 [0.98–1.29]	0.60 [0.47–0.94]^a^
50–75%	0.93 [0.81–1.06]	0.47 [0.34–0.66]^a^	0.74 [0.64–0.87]^a^	0.36 [0.26–0.54]^a^	0.48 [0.21–0.26]^a^	0.24 [0.17–0.35]^a^
75–100%	0.62 [0.56–0.69]^a^	0.55 [0.41–0.75]^a^	0.40 [0.36–0.46]	0.37 [0.26–0.52]^a^	0.24 [0.42–0.55]^a^	0.27 [0.19–0.38]^a^
**Fentanyl UDS groups (ref: 0–25%)**						
25–50%	2.23 [1.90–2.63]^a^	1.42 [0.96–2.03]^a^	2.32 [1.97–2.75]^a^	1.31 [0.90–1.90]	1.52 [1.31–1.78]^a^	1.24 [0.86–1.78]
50–75%	1.10 [0.96–1.25]	0.67 [0.48–0.92]^a^	0.77 [0.67–0.9]	0.55 [0.38–0.78]^a^	0.40 [0.35–0.46]^a^	0.31 [0.30–0.61]^a^
75–100%	0.58 [.52–.64]^a^	0.95 [0.69–1.29]	0.31 [0.27–0.36]	0.41 [0.28–0.61]^a^	0.10 [0.08–0.11]^a^	0.19 [0.17–0.38]^a^
**Cocaine UDS Groups (ref: 0–25%)**						
25–50%	1.31 [1.18–1.46]^a^	0.91 [0.69–1.2]	1.1 [0.98–1.23]	0.77 [0.57–1.03]	0.86 [0.78–0.94]^a^	0.74 [0.56–0.98]^a^
50–75%	0.97 [0.86–1.08]	0.82 [0.61–1.17]	0.70 0[.62–0.79]^a^	0.55 [0.40–0.76]^a^	0.44 [0.40–0.49]^a^	0.50 [0.36–0.69]^a^
75–100%	0.61 [.56–0.67]^a^	0.61 [0.45–.82]^a^	0.34 [0.31–0.38]^a^	0.32 [0.24–0.45]^a^	0.20 [0.18–0.22]^a^	0.25 [0.18–0.35]^a^
**Cannabis UDS Group (ref: 0–25%)**						
25–50%	5.71 [4.46–7.31]^a^	2.95 [1.62–5.39]^a^	10.15 [7.93–13.0]^a^	5.97 [3.27–10.90]^a^	13.53 [10.72–17.05]^a^	7.59 [4.20–13.71]^a^
50–75%	2.90 [2.45–3.43]^a^	1.04 [0.70–1.55]	3.96 [3.32–4.71]^a^	1.50 [0.99–2.27]	4.56 [3.91–5.32]^a^	1.93 [1.29–2.87]^a^
75–100%	2.57 [2.37–2.79]^a^	1.70 [1.34–2.14]^a^	3.71 [3.40–4.05]^a^	2.12 [1.64–2.72]^a^	4.26 [3.95–4.6]^a^	2.40 [1.89–3.04]^a^
**Other Opioid Groups (ref: 0–25%)**						
25–50%	0.78 [0.714–.86]^a^	0.64 [0.5–.82]^a^	0.54 [0.49–0.60]^a^	0.44 [0.33–0.57]^a^	0.34 [0.31–0.37]^a^	0.41 [0.31–0.53]^a^
50–75%	0.48 [0.42–.53]^a^	0.56 [0.40–0.77]^a^	0.23 [0.19–0.26]^a^	0.35 [0.24–0.51]^a^	0.12 [0.11–0.14]^a^	0.17 [0.12–0.26]^a^
75–100%	0.18 [0.15–0.21]^a^	0.33 [0.19–0.57]^a^	0.07 [0.197–0.26]	0.17 [0.09–0.36]^a^	0.03 [0.02–0.04]^a^	0.03 [0.01–0.09]^a^
**Starting dose above Median**						
	1.09 [1.64–1.91]^a^	1.08 [0.81–1.44]	2.30 [2.17–2.55]^a^	1.21 0[.90–1.60]	4.48 [4.18–4.80]^a^	1.40 [1.06–1.87]^a^
**Peak dose above the Median**						
	3.50 [3.38–3.84]^a^	0.87 [0.56–1.35]	4.97 [4.57–5.40]^a^	0.86 [0.54–1.34]	7.74 [7.20–8.33]^a^	0.90 [0.58–1.28]

*Abbreviations*: ^a^*aOR* adjusted odds ratio, *UDS* Urine Drug Screening

## Discussion

This study sought to describe OAT engagement and attrition trends using the cascade of care framework in Ontario between 2014 and 2020, characterize correlates associated with OAT retention at various stages along the continuum of care, and examine how retention is affected by the geographical location of residence. Drawing on longitudinal EMR data from a network of over 70 clinics across Ontario, we identified a distinct increasing number of patients in OAT from 2014 to 2020. Patients in the cohort who were retained longer in OAT tended to be younger, have a higher number of treatment attempts, have had a higher number of monthly UDS (indicating more frequent contact with an OAT clinic), and live in Northern and rural areas of Ontario. Whereas those patients who were using drugs such as fentanyl, amphetamines, cannabis, and cocaine while in treatment were less likely to be retained longer.

Our first objective was to describe the cascade of care for patients who have accessed OAT from a network of specialized addiction clinics in Ontario, Canada. We observed an increasing number of patients engaged in OAT over time. This finding was not surprising given the continuous effort to expand addiction care and treatment in Ontario in recent years, including ensuring access to OAT and reducing prescriber restrictions and requirements [22, 43, 44]. Particularly, in the era of fentanyl, OAT has become more accessible in an effort to reduce overdose deaths [44]. On the other hand, the increasing trend of OAT retention over time is supported by the literature on treatment cycling and re-attempts, which has indicated that restarting treatment has increased as the OAT program has expanded and access to treatment has increased [20]. However, as of 2020, still many patients with OUD were not adequately retained in treatment for 1 year or more. Lower retention rates in 2020 may be explained by the increase in buprenorphine/naloxone prescribing [45]. Several studies indicate that retention is lower for buprenorphine/naloxone compared to methadone patients [46]. Authors agree that Buprenorphine/naloxone is a safer agent. However, its advantage over methadone is tempered by the emerging evidence of problematic diversion and limited effectiveness for patients with more severe and chronic OUD [47]. This low retention rate indicates a need to enhance strategies to recruit new, potentially harder-to-reach patients with OUD who are not seeking treatment.

A number of previous opioid studies have employed a cascade of care framework to measure patient retention in OAT [24, 30]. However, there are multiple ways this framework has been operationalized, which makes it difficult to draw comparisons across these studies. For example, one Florida-based research group has conceptualized four different cascades of care which are: prevalence of OUD, diagnosed patients with OUD, initiation of treatment, and treatment continuation [30]. However, because of the nature of the database we used, we only had access to patients’ data who had accessed the OAT from one of the 70 clinics. Therefore, the prevalence of OUD who were not engaged in the OAT was beyond scope of this study. On the other hand, a British Columbia (BC), Canada-based study has used eight-stage cascades of care which are: OUD diagnosed, ever engaged in OAT, recently engaged in OAT, currently on OAT, retained in OAT ≥1 month, 3 months, 12 months, and 24 months [24]. This Canadian study identified significant attrition of rural patients from OAT whereas the current study identified that rural patients are more likely to be retained in the over 2 years cascades. On the other hand, in 2017, the BC study reported that 27% of patients were retained for over 1 year whereas in Ontario 61% of patients were retained for over 1 year in the OAT. The Florida and BC study both ended in 2017/2018. This is important to note because, in recent years in North America, the introduction of synthetic opioids such as fentanyl and other highly potent analogs has become exponentially more present in the drug supply has contributed to a rapidly worsening disease burden in recent years [1, 48–51]. In Canada, 4614 opioid-related deaths occurred in 2018, and nearly three-quarters of these deaths involved fentanyl or other synthetic opioids [52]. These contaminants are up to 10,000 times more potent than morphine [53, 54]. This is contributing to a mounting global public health concern [55] and may have contributed to the differences in retention in our study which included data from recent years compared to others. The retention differences between the Ontario and BC study may also be explained by the fact that the BC study reported the percentage of people with OUD retained for 1 year. Whereas this current study only reported the percentage of patients who accessed OAT who were retained for 1 year.

The second objective was to evaluate correlates associated with retention in OAT at various stages along the continuum of engagement. We found a certain degree of heterogeneity across the retention groups. Specifically, those with more treatment attempts and a higher number of monthly UDS were more likely to be retained for longer in OAT. This observation is likely reflective of the accumulation of OAT experience and more frequent and closer contact with OAT clinics. Similar findings have been supported in the OAT literature and the literature on smoking cessation [56, 57], indicating that the number of cessation or treatment attempts is a predictor of successful substance use treatment.

We found that continued drug use while in treatment, as measured by UDS results, was found to be associated with shorter retention. Recent reports have demonstrated an increase in the co-use of methamphetamine and opioids. The impact of poly-drug use on OAT retention patterns is well-documented in the literature [19, 58–61]. Our study findings offer further evidence that poly-drug use impacts patients’ OAT engagement at various stages. With an increase in poly-substance use, it is, therefore, likely that fewer people will be retained in OAT at a time when they may be at heightened risk in the era of enhanced exposure to potent opioid analogs such as fentanyl. Accordingly, efforts for tailored interventions or alternative interventions for patients who continue to use drugs while in treatment may improve treatment retention.

The third objective was to assess the impact of patients’ location of residence on retention in OAT. We found that when adjusted covariates, relative to the Southern urban areas of Ontario, patients living in Northern rural areas were more likely to be retained in OAT for more than 2 years. This association with patients’ location of residence is important because it shows the regional variation in OAT retention within various cascades. Interestingly, despite the well-known barriers to accessing addiction and related services in rural areas due to limited access to providers and long distances to travel [31, 59, 62], our findings are also reflected in a previous Ontario based-study which found that OAT patients in Northern regions (both urban and rural) were more likely to be retained in methadone maintenance therapy compared to the Southern regions. The higher retention in Northern Rural patients can be explained under the presumption that patients who overcome the barriers to accessing treatment in rural areas may have higher motivation for treatment [31, 63]. Notably, the study by Eibl et al., uses administrative data from 2003 to 2012 in contrast to our study timeline which is 2014–2020, which strengthens the evidence-base relating to geography and OAT.

Some limitations in the current study require consideration. Firstly, this study used a cohort of patients receiving OAT from a network of clinics in Ontario. We estimate based on previously published data on all OAT patients in Ontario [64, 65], that this study’s cohort encompasses approximately 50% of OAT patients in Ontario. Secondly, we did not have access to data for patients who were currently not engaged in OAT. Having access to this data would allow us to compare the characteristics of the people with OUD who were presently engaged within multiple cascades with those who were not engaged at all. Thirdly, we did not have access to important personal characteristics that may have modified the association between patient retention and their characteristics, such as other concurrent disorders, history of homelessness, mental health diagnoses, or other psychosocial factors [24, 29]. Finally, the choice of starting medication was made based on clinical characteristics and patient choice at the time of treatment initiation and was not randomly assigned. Therefore, differences in retention correlated with starting medication may reflect differences in the patient characteristics for whom methadone was chosen rather than a difference in the effectiveness of the medication relative to buprenorphine/naloxone. Similarly, because of the way the database was set up, we were unable to track medication switches and we were only able to track what medication patients started on their first treatment window.

## Conclusion

In conclusion, through the application of the cascade of care framework, we identified a distinct increase in retention in OAT from 2014 to 2020. We also observed various individual-level and clinical level factors associated with retention at various stages, including regional variation in OAT retention. This finding highlights the potential value of acquiring a better understanding of patients’ long-term OAT retention patterns and the associated impacts of such patterns on OAT outcomes. Our findings further suggest a need for more research at various stages of the OAT continuum to maximize the benefits of OAT.

## Data Availability

The datasets presented in this article are not readily available because the datasets contain identifiable confidential patient information and cannot be shared with anyone not approved by the Laurentian University Research Ethics Board. In other words, ethics approvals stipulate restrictions that apply to the availability of these data. Additional data enquiries are welcomed by the authors of this paper. For additional inquiry, please contact, farah.tahsin@mail.utoronto.ca.

## Abbreviations

OAT: Opioid Agonist Treatment
OUD: Opioid Use Disorder
ODB: Ontario Drug Benefits
EMR: Electronic Medical Records
CATC: The Canadian Addiction Treatment Centers
Strobe: Strengthening the Reporting of Observational Studies in Epidemiology
OMA: The Ontario Medical Association
RIO: Rurality Index of Ontario
API: application program interface
LHIN: Local Health Integration Network
UDS: Urine Drug Screening
BUP: Buprenorphine
aOR: Adjusted Odds Ratio
SD: Standard Deviation

## References

[1] Belzak L, Halverson J (2018). Evidence synthesis - The opioid crisis in Canada: a national perspective. Health Promot Chronic Dis Prev Can.

[2] Special Advisory Committee on the Epidemic of Opioid Overdoses. Opioid- and Stimulant-Related Harms in Canada - Public Health Infobase | Public Health Agency of Canada. 2021. https://health-infobase.canada.ca/substance-related-harms/opioids-stimulants/graphs?index=477. Accessed 27 Sept 2021.

[3] Williams AR, Nunes EV, Bisaga A, Levin FR, Olfson M (2019). Development of a Cascade of Care for Responding to the Opioid Epidemic. Am J Drug Alcohol Abuse.

[4] Yarborough BJH, Stumbo SP, McCarty D, Mertens J, Weisner C, Green CA (2016). Methadone, buprenorphine and preferences for opioid agonist treatment: A qualitative analysis. Drug Alcohol Depend.

[5] Amiri S, Hirchak K, Lutz R (2018). Three-year retention in methadone opioid agonist treatment: A survival analysis of clients by dose, area deprivation, and availability of alcohol and cannabis outlets. Drug Alcohol Depend.

[6] Bharat C, Degenhardt L, Dobbins T, Larney S, Farrell M, Barbieri S (2021). Using administrative data to predict cessation risk and identify novel predictors among new entrants to opioid agonist treatment. Drug Alcohol Depend.

[7] Clark RE, Baxter JD, Aweh G, O’Connell E, Fisher WH, Barton BA (2015). Risk Factors for Relapse and Higher Costs Among Medicaid Members with Opioid Dependence or Abuse: Opioid Agonists, Comorbidities, and Treatment History. J Subst Abus Treat.

[8] Samples H, Williams AR, Olfson M, Crystal S (2018). Risk factors for discontinuation of buprenorphine treatment for opioid use disorders in a multi-state sample of Medicaid enrollees. J Subst Abus Treat.

[9] Degenhardt L, Bucello C, Mathers B (2011). Mortality among regular or dependent users of heroin and other opioids: a systematic review and meta-analysis of cohort studies. Addiction.

[10] Morin KA, Parrotta MD, Eibl JK, Marsh DC (2011). A Retrospective Cohort Study Comparing In-Person and Telemedicine-Based Opioid Agonist Treatment in Ontario, Canada, Using Administrative Health Data. Res Article Eur Addict Res.

[11] About Us | Canadian Addiction Treatment Centres. https://canatc.ca/about-us/. Accessed 30 Jan 2022.

[12] Rural and Northern Community Issues in Mental Health. https://ontario.cmha.ca/documents/rural-and-northern-community-issues-in-mental-health/. Accessed 11 Dec 2021.

[13] Bruneau J, Ahamad K, Goyer MÈ (2018). Management of opioid use disorders: A national clinical practice guideline. CMAJ..

[14] Dorman K, Biedermann B, Linklater C, Jaffer Z (2018). Community strengths in addressing opioid use in Northeastern Ontario. Can J Public Health.

[15] Canadian Medical Association (2000). Rural and remote practice issues. CMAJ..

[16] Canadian Medical Association. Rural and Northern Community Issues in Mental Health, vol. 163; 2009. https://ontario.cmha.ca/documents/rural-and-northern-community-issues-in-mental-health/. Accessed 13 Feb 2022.

[17] Hartley D (2004). Rural health disparities, population health, and rural culture. Am J Public Health.

[18] Peles E, Linzy S, Kreek MJ, Adelson M (2008). One-Year and Cumulative Retention as Predictors of Success in Methadone Maintenance Treatment: A Comparison of Two Clinics in the United States and Israel.

[19] Stone AC, Carroll JJ, Rich JD, Green TC (2020). One year of methadone maintenance treatment in a fentanyl endemic area: Safety, repeated exposure, retention, and remission.

[20] Bell J, Burrell T, Indig D, Gilmour S (2006). Cycling in and out of treatment; Participation in methadone treatment in NSW, 1990-2002. Drug Alcohol Depend.

[21] Sordo L, Barrio G, Bravo MJ (2017). Mortality risk during and after opioid substitution treatment: systematic review and meta-analysis of cohort studies. BMJ (Clinical research ed).

[22] Ontario Drug Policy Network (2018). Landscape of Prescription Opioid Use: Patterns, Trends and Geographic Variation in Ontario, Canada.

[23] Gardner EM, MP ML, Steiner JF, Del Rio C, Burman WJ (2011). The spectrum of engagement in HIV care and its relevance to test-and-treat strategies for prevention of HIV infection. Clin Infect Dis.

[24] Piske M, Zhou H, Min JE (2020). The cascade of care for opioid use disorder: a retrospective study in British Columbia, Canada. Addiction.

[25] Ali M, Bullard K, Gregg E, del Rio C (2014). A cascade of care for diabetes in the United States: visualizing the gaps. Ann Intern Med.

[26] Safreed-Harmon K, Blach S, Aleman S (2019). The Consensus Hepatitis C Cascade of Care: Standardized Reporting to Monitor Progress Toward Elimination. Clin Infect Dis.

[27] Janjua NZ, Kuo M, Yu A (2016). The Population Level Cascade of Care for Hepatitis C in British Columbia, Canada: The BC Hepatitis Testers Cohort (BC-HTC). EBioMedicine..

[28] Williams A, Nunes E, Olfson M (2017). To Battle The Opioid Overdose Epidemic, Deploy The ‘Cascade Of Care’ Model.

[29] Krebs E, Min JE, Zhou H, Davison C, McGowan G, Nosyk B (2021). The cascade of care for opioid use disorder among youth in British Columbia, 2018. J Subst Abus Treat.

[30] Johnson K, Hills H, Ma J, Brown CH, McGovern M (2021). Treatment for opioid use disorder in the Florida medicaid population: Using a cascade of care model to evaluate quality. Am J Drug Alcohol Abuse.

[31] Eibl JK, Gomes T, Martins D (2015). Evaluating the Effectiveness of First-Time Methadone Maintenance Therapy Across Northern, Rural, and Urban Regions of Ontario, Canada. J Addict Med.

[32] Morin KA, Prevost C, Eibl JK, Franklyn MT, Moise A, Marsh D. A retrospective cohort study evaluating correlates of deep tissue infections among patients enrolled in opioid agonist treatment using administrative data in Ontario, Canada. PLoS One. 2020;15(4). 10.1371/JOURNAL.PONE.0232191. 10.1371/journal.pone.0232191 PMC7182261 32330184

[33] Elm E von, Altman DG, Egger M, et al. The Strengthening the Reporting of Observational Studies in Epidemiology (STROBE) Statement: Guidelines for Reporting Observational Studies. PLoS Med 2007;4(10):e2e96. 10.1371/JOURNAL.PMED.0040296. 10.1371/journal.pmed.0040296 PMC2020495 17941714

[34] British Columbia Centre on Substance Use (BCCSU). A Guideline for the Clinical Management of Opioid Use Disorder. 2017. http://www.bccsu.ca/care-guidance-publications/. Accessed 13 Feb 2022.

[35] Peles E, Schreiber S, Adelson M (2006). Factors predicting retention in treatment: 10-year experience of a methadone maintenance treatment (MMT) clinic in Israel. Drug Alcohol Depend.

[36] O’Connor AM, Cousins G, Durand L, Barry J, Boland F (2020). Retention of patients in opioid substitution treatment: A systematic review. PLoS One.

[37] Timko C, Schultz NR, Cucciare MA (2019). Retention in medication-assisted treatment for opiate dependence: A systematic review. J Addict Dis.

[38] RIO Postal Code Look-up. https://apps.oma.org/RIO/index.html. Accessed 25 Oct 2021.

[39] Local Health System Integration Act, 2006, S.O. 2006, c. 4. https://www.ontario.ca/laws/statute/06l04. Accessed 30 Jan 2022.

[40] Eibl JK, Gauthier G, Pellegrini D (2017). The effectiveness of telemedicine-delivered opioid agonist therapy in a supervised clinical setting. Drug Alcohol Depend.

[41] Franklyn A, Franklyn AM, Eibl JK, Lightfoot NE (2017). Geography, Treatment Modality , and Substance Use : Evaluating Factors That Impact Opioid Agonist Therapy in Northern Ontario , Canada. J Addict Med Ther.

[42] Aird P, Kerr J. Factors Affecting Rural Medicine: an improvement on the Rurality Index of Ontario. Can J Rural Med. 2007;12(4):245–7. https://go.gale.com/ps/i.do?p=AONE&sw=w&issn=12037796&v=2.1&it=r&id=GALE%7CA170196095&sid=googlScholar&linkaccess=fulltext. Accessed 25 Oct 2021. 18076819

[43] College of Physicians and Surgeons of Ontario (2015). Paper presented at: CPSO Methadone Conference.

[44] Dong H, Hayashi K, Fairbairn N, et al. Long term pre-treatment opioid use trajectories in relation to opioid agonist therapy outcomes among people who use drugs in a Canadian setting. Addict Behav. 2021;112. 10.1016/J.ADDBEH.2020.106655. 10.1016/j.addbeh.2020.106655 PMC7572870 32977270

[45] Whelan P, Remski K (2012). Buprenorphine vs methadone treatment: A review of evidence in both developed and developing worlds. J Neurosci Rural Pract.

[46] Petitjean S, Stohler R, Déglon JJ (2001). Double-blind randomized trial of buprenorphine and methadone in opiate dependence. Drug Alcohol Depend.

[47] Mariolis T, Bosse J, Martin S, Wilson A, Chiodo L (2019). A Systematic Review of the Effectiveness of Buprenorphine for Opioid Use Disorder Compared to Other Treatments: Implications for Research and Practice. J Addict Res Ther.

[48] British Columbia Coroners Service (2019). Fentanyl-Detected Illicit Drug Overdose Deaths: January 1, 2012 to January 31, 2019.

[49] Zoorob M (2019). Fentanyl shock: The changing geography of overdose in the United States. Int J Drug Policy.

[50] Gomes T, Tadrous M, Mamdani MM, Paterson JM, Juurlink DN (2018). The Burden of Opioid-Related Mortality in the United States. JAMA Netw Open.

[51] Health TLP (2018). A public health approach to Canada’s opioid crisis. Lancet Public Health.

[52] Health Canada. Overview of national data on opioid-related harms and deaths. 2018 Available from: https://www.canada.ca/en/health-canada/services/substance-use/problematic-prescription-drug-use/opioids/datasurveillance-research/harms-deaths.html. Retrieved on January 2, 2020.

[53] Fairbairn N, Coffin PO, Walley AY (2017). Naloxone for heroin, prescription opioid, and illicitly made fentanyl overdoses: Challenges and innovations responding to a dynamic epidemic. Int J Drug Policy.

[54] Misailidi N, Papoutsis I, Nikolaou P, Dona A, Spiliopoulou C, Athanaselis S (2018). Fentanyls continue to replace heroin in the drug arena: the cases of ocfentanil and carfentanil. Forensic Toxicol.

[55] United Nations Office on Drugs and Crime (2019). World Drug Report.

[56] Hellman R, Cummings KM, Haughey BP, Zielezny MA, O’Shea RM (1991). Predictors of attempting and succeeding at smoking cessation. Health Educ Res.

[57] won Lee C, Kahende J. Factors Associated With Successful Smoking Cessation in the United States, 2000. Am J Public Health 2007;97(8):1503. 10.2105/AJPH.2005.083527. 10.2105/AJPH.2005.083527 PMC1931453 17600268

[58] Franklyn AM, Eibl JK, Gauthier GJ, Pellegrini D, Lightfoot NE, Marsh DC (2017). The impact of cocaine use in patients enrolled in opioid agonist therapy in Ontario, Canada. Intern J Drug Policy.

[59] Franklyn AM, Eibl JK, Gauthier G, Pellegrini D, Lightfoot NK, Marsh DC. The impact of benzodiazepine use in patients enrolled in opioid agonist therapy in Northern and rural Ontario. Harm Reduct J. 2017;14(1). 10.1186/s12954-017-0134-5. 10.1186/s12954-017-0134-5 PMC5267412 28122579

[60] Franklyn AM, Eibl JK, Gauthier GJ, Marsh DC. The impact of cannabis use on patients enrolled in opioid agonist therapy in Ontario, Canada. PLoS ONE. 2017;12(11). 10.1371/journal.pone.0187633. 10.1371/journal.pone.0187633 PMC5678697 29117267

[61] Morin KA, Vojtesek F, Acharya S, Marsh DC (2021). Negative Impact of Amphetamine-Type Stimulant Use on Opioid Agonist Treatment Retention in Ontario, Canada. Front Psychiatry.

[62] Kiepek N, Hancock L, Toppozini D, Cromarty H, Morgan A, Kelly L. Facilitating medical withdrawal from opiates in rural Ontario. Rural Remote Health. 2012;12(4). 10.22605/RRH2193. 23094953

[63] Li JX, McMahon LR, Gerak LR, Becker GL, France CP (2008). Interactions between Delta(9)-tetrahydrocannabinol and mu opioid receptor agonists in rhesus monkeys: discrimination and antinociception. Psychopharmacology..

[64] Morin KA, Eibl JK, Gauthier G (2020). A cohort study evaluating the association between concurrent mental disorders, mortality, morbidity, and continuous treatment retention for patients in opioid agonist treatment (OAT) across Ontario, Canada, using administrative health data. Harm Reduct J.

[65] Interactive Opioid Tool | Public Health Ontario. https://www.publichealthontario.ca/en/data-and-analysis/substanceuse/interactive-opioid-tool. Accessed 4 Nov 2021.

